# Presenting symptoms in children with neurofibromatosis type 2

**DOI:** 10.1007/s00381-020-04729-w

**Published:** 2020-06-15

**Authors:** Isabel Gugel, Florian Grimm, Christian Teuber, Julian Zipfel, Marcos Tatagiba, Victor-Felix Mautner, Martin Ulrich Schuhmann, Lan Kluwe

**Affiliations:** 1 Department of Neurosurgery, University Hospital Tübingen, Tübingen, Germany; 2 Centre of Neurofibromatosis, Centre of Rare Disease, University Hospital Tübingen, Tübingen, Germany; 3 Division of Pediatric Neurosurgery, University Hospital Tübingen, Tübingen, Germany; 4 Department of Neurology, University Medical Center Hamburg-Eppendorf, Hamburg, Germany; 5 Department of Maxillofacial Surgery, University Medical Center Hamburg-Eppendorf, Hamburg, Germany

**Keywords:** Neurofibromatosis type 2, Vestibular schwannoma, Presenting symptom, Pediatric

## Abstract

**Purpose:**

The hallmark of neurofibromatosis type 2 (NF2) is the presence of bilateral vestibular schwannomas (VS) which however have not yet developed or grown to large size in children and young adolescents. Therefore, early diagnosis in pediatric patients without family history of NF2 has to be made by signs and symptoms not related to VS which will be reviewed in this study.

**Methods:**

A total of 70 children diagnosed for NF2 at an age of < 18 years were identified from our patient cohort. Age and symptoms, signs and pathology at symptom onset, age at NF2 diagnosis and symptoms leading to diagnosis as well as genetic findings were retrospectively reviewed.

**Results:**

The average age at symptom/sign onset was 8 ± 6 (range 0–17) years and 11 ± 5 (range 1–17) years at time of diagnosis. Fifteen children had a positive family history and were diagnosed upon additional clinical symptoms. The most frequent first presenting symptom/signs were ophthalmological abnormalities (49%), followed by cutaneous features (40%), non-VS-related neurological deficits (33%), and symptoms attributable to VS (21%). VS were not only the most common symptomatic neoplasm but also the most frequent pathological evidence for the diagnosis (72%). In 42 patients with available genetic testing results, pathogenic mutations were most frequently identified (*n* = 27).

**Conclusion:**

The presenting symptoms in NF2 children appear “unspecific” or less specific for classical NF2 compared with adult NF2 patients, posing a challenge particularly for cases without family history. In children, ophthalmological and cutaneous features should raise clinical suspicion for NF2 and referral to an NF2 specialized center is recommended.

## Introduction

**Fig. 1:**
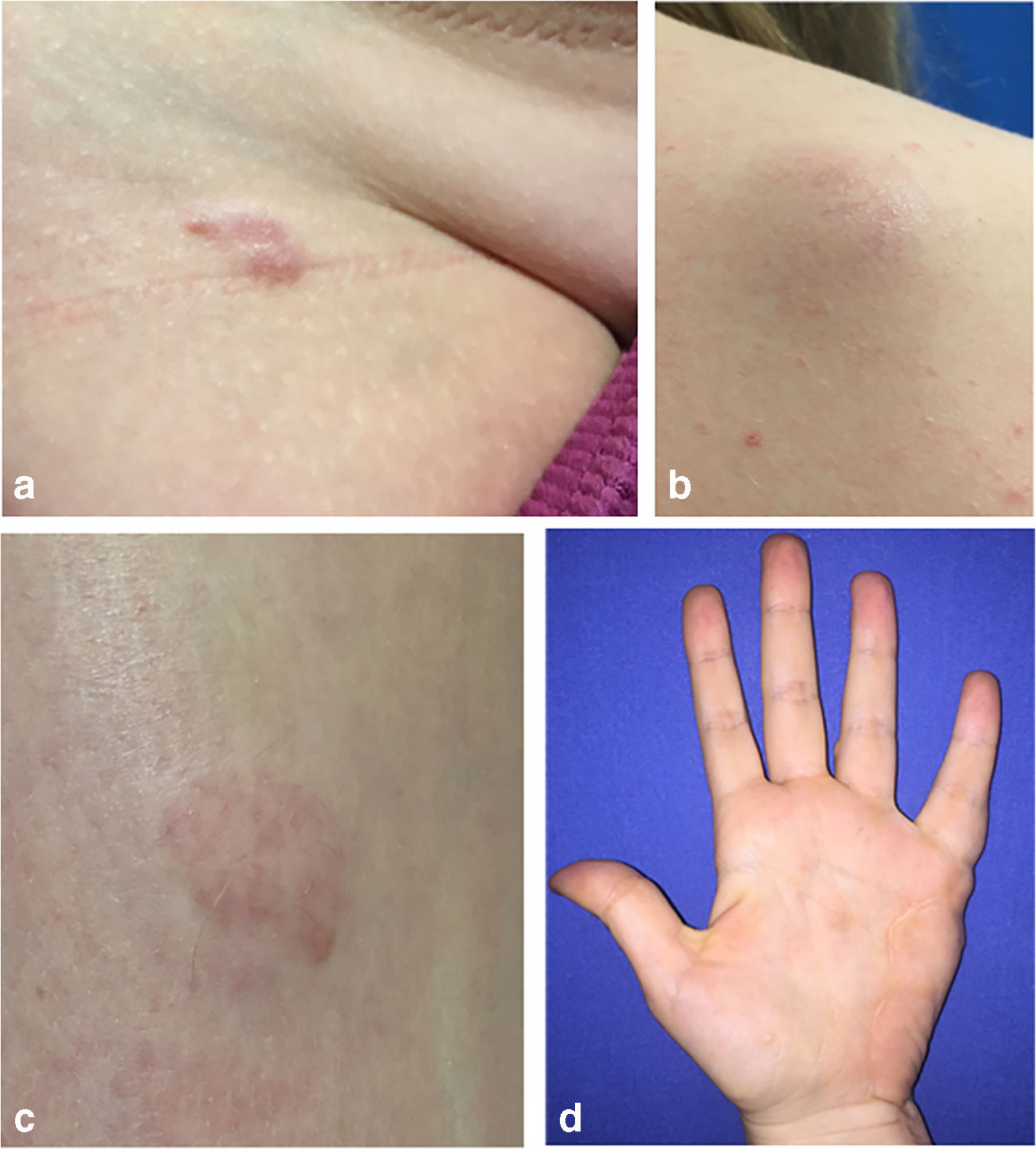
**a**–**d** Typical cutaneous features in children and early adolescents with NF2. **a** Intracutaneous schwannoma of the groin in an 8-year-old girl. **b** Predominantly subcutaneous, large schwannoma of the scapula of a 16-year-old female patient. **c** Intracutaneous schwannoma on the lower leg of the same patient (patient **b**). **d** Plexiform schwannoma of the ulnar nerve left-sided in a 19-year-old girl who previously received surgery at 13 years of age

**Fig. 2:**
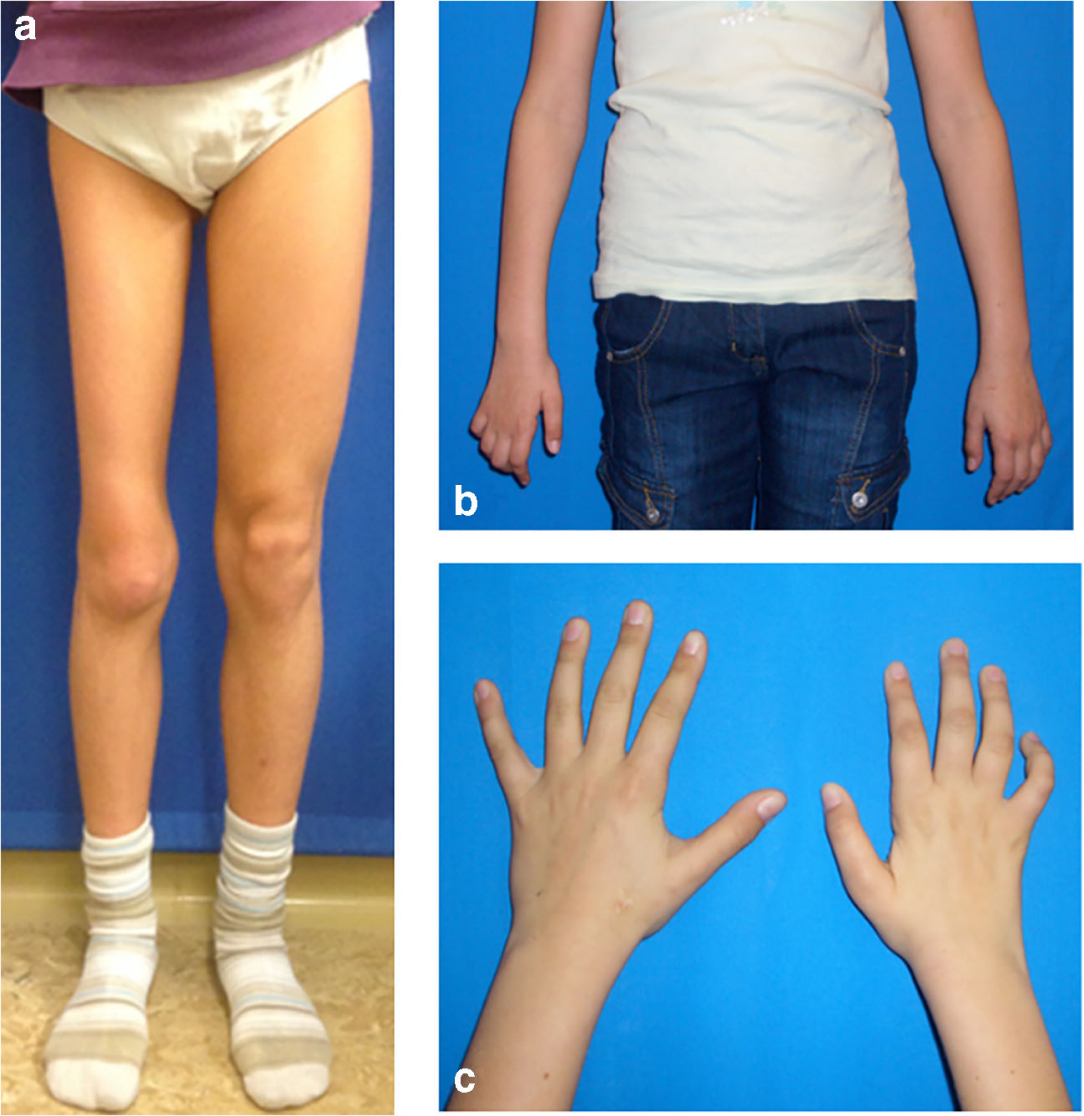
**a**–**c** Examples of NF2-associated neuropathy. **a** NF2-associated neuropathy of the right leg with muscle atrophy in an 8-year-old patient. **b**, **c** Muscle atrophy of the right hand due to NF2-associated neuropathy of the right median and ulnar nerves as well as shortening of the arm as presenting symptoms in a 10-year-old patient

The genetic tumor predisposition syndrome neurofibromatosis type 2 (NF2) is a rare disease with a prevalence of 1:56.000 and an incidence of 1 case in 33,000 to 40,000 live births per year [1, 2]. It is caused by the inactivation of the *NF2* tumor suppressor gene located on chromosome band 22q12.2 [3, 4]. Patients exhibit benign tumors of the central and peripheral nervous system (e.g., schwannomas, meningiomas, ependymomas) and other non-tumorous findings such as ocular abnormalities (e.g., cataract, retinal hamartoma, epiretinal membranes), cutaneous manifestations (e.g., skin tumors, Fig. 1) [5, 6], or muscle atrophy due to NF2-associated neuropathy (Fig. 2) [6–8]. The typical hallmark of the disease which also secures the diagnosis is bilateral vestibular schwannomas (VS) causing tinnitus, balance disorders, dizziness, and later hearing loss and facial palsy depending on tumor size [5]. Total deafness is an expectable problem over lifetime in these patients, which can result in serious social and educational problems. Therefore, independent of treatment modality choice, long-term hearing preservation should be the major goal in these patients. To pursue this goal, early diagnosis with ensuing early awareness of VS and monitoring of hearing is crucial.

While older adolescents and adults usually present with symptoms attributable to VS (e.g., hearing loss, tinnitus, balance disorder, facial palsy), children and young adolescents commonly present with a variety of symptoms unrelated to VS [9–13] including ophthalmological and cutaneous findings. Also unspecific symptoms and pathologies such as vascular events can be the first symptom of NF2 [14]. Consecutively, diagnosis for NF2 may be largely delayed with the consequence of wasting the valuable time-window for monitoring VS tumor growth and hearing in the early stage.

In this study, we retrospectively reviewed our NF2 cohort diagnosed at age younger than 18 years, focusing on the variety of very first symptoms which initialized further examination resulting in the final diagnosis of NF2.

## Material and methods

### Patient cohort

For this monocentric analysis, we retrospectively reviewed medical records of 70 patients who were diagnosed, monitored, or treated in our Centre for Neurofibromatosis between 2003 and 2019 and at an age younger than 18 years at time of diagnosis. The Ethics Board of the Medical Faculty and the University Hospital of Tübingen approved this retrospective analysis (No. 018/2019BO2).

Diagnosis of NF2 was based on clinical, radiological (cranial and spinal magnetic resonance imaging (MRI)), and ophthalmological (with the focus on NF2-associated features) examination using the established criteria [15].

After confirmation of diagnosis, a strict and frequent hearing monitoring (pure-tone and speech audiometry, as well as brainstem auditory, evoked potentials) as previously described was completed [16]. Mutation analysis of the *NF2 gene* could be performed in 42 patients. The other 28 patients or their parents did not wish or agree for a genetic test.

We distinguished between symptom(s)/signs/pathology(ies) at symptom onset (= presenting symptoms) and those leading to diagnosis. The former are often non-specific for NF2 and partially do not count as true diagnostic criteria.

Significance of comparisons between groups was determined by *t* test with a two-tailed hypothesis and significance level was set to *p* < 0.05.

## Results

Detailed demographic, clinical, and genetic data are given in Tables 1, 2, 3, and 4.

**Table 1:** Demographic data of 70 neurofibromatosis type 2 (NF2) patients with age < 18 years at diagnosis

Family history	All	Positive	Negative
No of patients/tumors	70	15	55
Sex (No of female/male)	37/33	5/10	32/23
Age at in years (mean ± SD, range)			
- symptom onset - diagnosis	8 ± 6, 0–17 11 ± 5, 1–17	11 ± 5, 0–17 12 ± 4, 3–17	8 ± 5, 0–17 11 ± 5, 1–17
Latency to diagnosis in months (mean ± SD, range)			
- all patients - symptoms at onset leading to diagnosis - symptoms at onset not leading to diagnosis Statistical significance	34 ± 50, 0-181 23 ± 43, 0-181, n = 48 68 ± 55, 0-173, n = 18 *p = 0.285*	13 ± 33, 0-129. n = 15 13 ± 33, 0-129, n = 13* -, n = 0 -	39 ± 52, 0-181 27 ± 45, 0-181, n = 35* 68 ± 55, 0-173, n = 18 *p = 0.491*
Detected mutation types (No of patients)	In 38 patients	in 11 patients	in 27 patients
- splicing mutations - nonsense mutations - frameshifting mutations - large genome alteration - missense mutation - large deletion - duplication	10 8 13 1 0 5 1	2 0 6 1 0 1 1	8 8 7 0 0 4 0

*No*, number; *SD*, standard deviation. *Asymptomatic patients who were diagnosed due to their positive family history or incidentally after trauma were excluded

**Table 2:** Details of presenting symptoms/signs of 70 children with NF2

Presenting symptom/features	No of patients (%)
Ocular/vision problem -Visual impairment/loss*^a^ -Strabismus*^b^ VS-related symptoms -Hypacusis -Sudden hearing loss -Tinnitus -Balance disorders -Dizziness -Hoarseness of voice -Facial nerve palsy (due to VS) Non-VS-related neurological symptoms -Seizures -Motor deficits -Sensory deficits -Dysarthria -Facial nerve palsy (non-VS related) -Back-/neck-/radiating pain -Proptosis Monosymptomatic/polysymptomatic -Monosymptomatic patients -Polysymptomatic patients (≥ 2 symptoms) -Asymptomatic	34 (49%) 15 19 15 (21%) 10 0 1 1 1 1 1 23 (33%) 3 9 2 1 2 3 3 28 (40%) 38 (54%) 4 (6%)

Values are numbers of patients. *VS*, vestibular schwannoma

*^a^Visual impairment was tumor associated in 4 patients due to optic nerve sheath meningiomas, in 3 patients due to intra/periorbital meningiomas/schwannomas. In the remaining 8 patients, the visual impairment or was attributed to cataracts or retinal hamartomas/maculopathy

*^b^Strabismus was idiopathic in 15 cases and tumor associated in 4 cases

**Table 3:** Causal pathology for presenting symptom/feature

Pathology/feature	No of patients
Cutaneous plexiform schwannoma	27 (39%)
Vestibular schwannoma	15 (21%)
Intracranial meningioma	8 (15%)
Cataract	6
Retinal hamartoma/maculopathy	5
Neuropathy/muscle atrophy	5
Peripheral nerve schwannoma*^a^	4
Intracranial non-VS schwannoma	4
Cerebral dysplasia	2
Café-au-lait spots	1
Bifrontal angiomatosis	1
Intracranial astrocytoma	1
Spinal meningioma	1
Spinal ependymoma	1
Ischemic brainstem stroke	1
SAH bleeding due to aneurysm	1

*^a^Subcutaneous or intramuscular peripheral schwannoma which were palpable or visible for the patients/examiner

**Table 4:** Discrepancy between symptoms/signs at symptom onset and symptoms leading to diagnosis in 18 patients

	Age in years			Symptoms/signs (pathologies)	
Patient ID	At symptom onset	At diagnosis	Latency in months	At symptom onset	Leading to diagnosis
1	17	17	0	Idiopathic strabismus, slight facial palsy (VS related)	Headache, dizziness and progressive facial palsy (large VS)
3	8	12	48	Slight facial palsy (VS related)	Balance disorders (VS related)
5	1	6	152	Cataract, epiretinal gliosis, muscle weakness (non-palpable peripheral nerve schwannoma)	Cutaneous plexiform schwannoma
8	14	15	12	Cutaneous plexiform schwannoma, asymptomatic and palpable subcutaneous/intramuscular peripheral nerve schwannoma, tumor-associated strabismus (schwannoma 3rd cranial nerve)	Hypacusis, balance disorders, tinnitus (all VS related)
9	10	15	60	Cutaneous plexiform schwannoma	Hypacusis (VS related), headache
13	4	13	102	Cutaneous plexiform schwannoma	Sensory deficits, back pain (spinal schwannomas)
14	10	13	36	Amblyopia, visual loss (subependymal astrocytoma lateral ventricle)	Cutaneous plexiform schwannoma
15	7	8	14	Cutaneous plexiform schwannoma	Visual impairment (retinal hamartoma)
26	6	16	120	Muscle weakness (neuropathy)	Hypacusis (VS related)
27	7	7	0	Muscle atrophy, visual loss, idiopathic strabismus	Hemiparesis, dysarthria (brainstem stroke)
31	5	14	107	Cutaneous plexiform schwannoma	Dizziness, tinnitus (VS related)
36	15	15	1	Cutaneous plexiform schwannoma, idiopathic strabismus, café-au-lait spots	Facial palsy (VS related)
37	5	12	84	Neuropathic pain (subcutaneous/intramuscular peripheral nerve schwannoma)	Hypacusis (VS related)
46	4	11	84	Idiopathic strabismus	Hypacusis, dizziness (VS related)
48	5	7	24	Idiopathic strabismus	Hypacusis (VS related)
50	1	13	132	Visual impairment (cataract), idiopathic strabismus	Dizziness, balance disorders, hypacusis, facial palsy, dysarthria (combined VS related and brainstem stroke)
53	1	15	173	Cutaneous plexiform schwannoma	Dizziness, hypacusis (VS related)
54	5	6	12	Facial palsy (VS related), cutaneous plexiform schwannoma, amblyopia, epiretinal gliosis, maculopathy	Hypacusis (VS related)

*VS*, vestibular schwannoma. All presenting symptoms which were tumor associated were initially not clarified by magnetic resonance imaging. In parentheses, the causative pathology is described

*NF2* mutations were found in 38 (90%) of the 42 tested patients, resulting in a very high frequency of positive genetic examination. One patient was genetically ascertained to be mosaic with a nonsense mutation found in tumor DNA. Only in three patients, no *NF2* mutations were found neither in tumor nor in blood DNA.

### Patients with a concordance between presenting symptoms/signs/pathologies and symptoms/signs/pathologies leading to diagnosis

In the vast majority (*n* = 52, 74%) of cases, the presenting symptoms corresponded to symptoms leading to diagnosis. In this group, the final diagnosis was established within 1 year in 32/52 (62%) patients. For the other 18 children, the NF2 diagnosis was secured more than 1 year later. Among these cases with delayed diagnosis, 16 patients suffered from ophthalmological abnormalities and 14 patients exhibited cutaneous features (examples are given in Fig. 1). However, regardless of latency, no additional symptoms or signs were instrumental for initializing the diagnostic process at a later time. Altogether, ophthalmological abnormalities were the most common presenting symptoms (49%), followed by cutaneous features (40%), non-VS-related neurological symptoms (33%), and last VS-related symptoms (21%).

### Presenting symptoms/finding not equal to those initializing the diagnosis process

In the remaining 18 patients of the cohort (26%), the first presenting symptoms and pathologies did not lead to further comprehensive examinations. Examinations resulting in the diagnosis of NF2 were initialized later upon newly developed symptoms/findings which were attributed to VS in most cases (13 = 72%).

### Late diagnosis in patients with a family history

Only two of 15 patients with a positive familiy history for NF2 were diagnosed early due to this fact. For the other 13 cases, no regular monitoring was recorded despite the fact that almost half of them had been presented to medical institutions before they became symptomatic. Consequently, the age at diagnosis was even 1 year later compared with patients without a family history. However, this difference was not significant (*p* > 0.05).

## Discussion

The clinical presentation in patients with neurofibromatosis type 2 is complex and variable, particularly in the pediatric age group. This group usually comes into medical attention with features not related to vestibular schwannoma (VS) but which are rather non-specific and this most often not right away recognized as feature pointing to NF2 in the differential diagnosis.

The majority of our patients exhibited ophthalmological (49%) complaints/abnormalities and skin features (40%, examples are illustrated in Fig. 1), followed by non-VS-related (33%) and last VS-related (21%) neurological symptoms. This clinical spectrum largely differs from that of later adolescents and adult NF2 patients who commonly present with symptoms attributable to VS [12, 15, 17].

Studies almost 30 years ago reported these findings that non-VS symptoms can be detected a young age and lead to early diagnosis [6]; however, these observations have not made it into common knowledge of pediatricians, pediatric neurosurgeons, and pediatric neurologists, as this often can be observed in rare disease, where the individual physician outside of centers only sees very few patients, if at all, during his career.

**Fig. 3:**
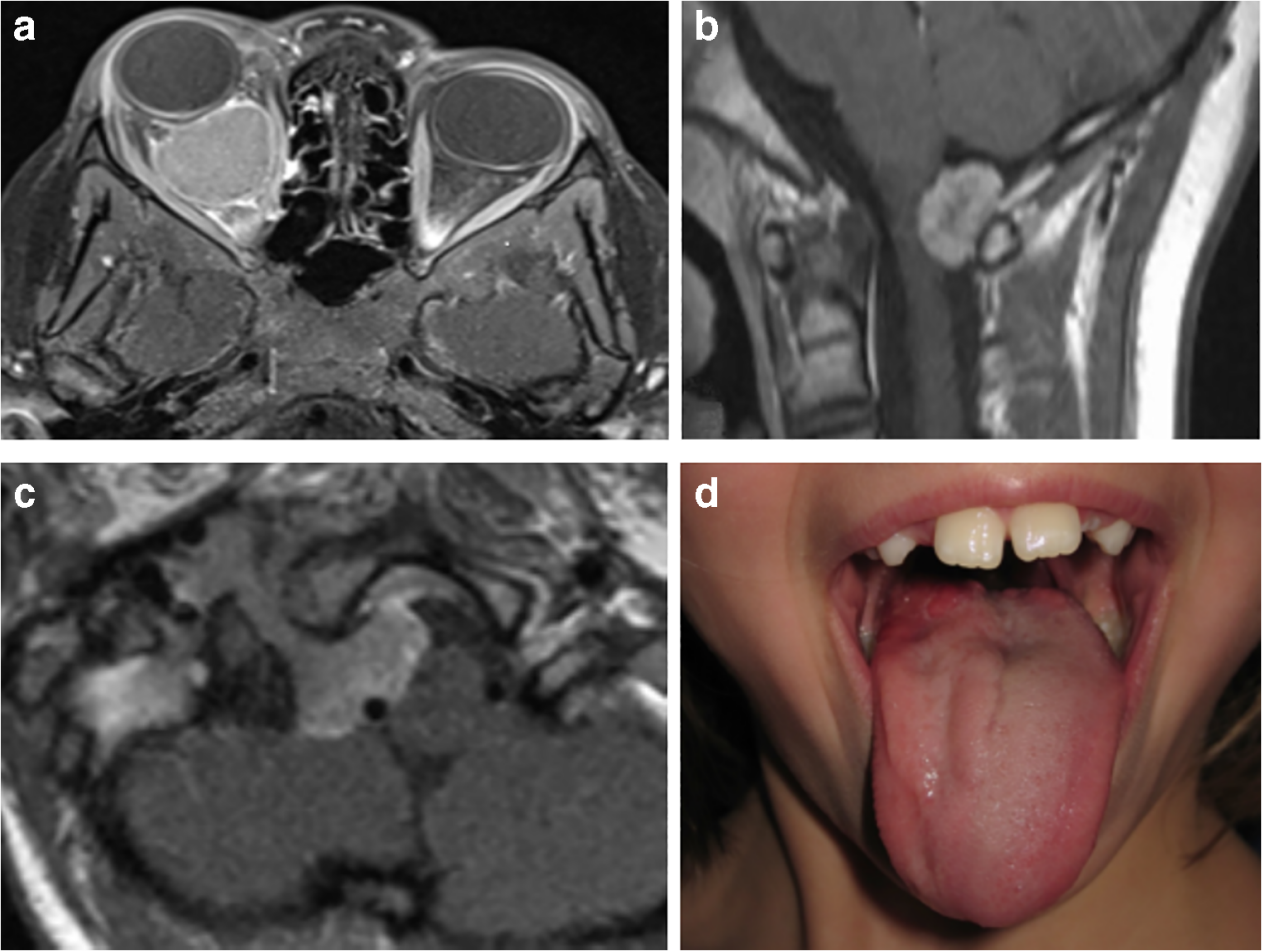
**a**–**d** Examples of NF2-associated intracranial meningioma manifestations. **a** Symptomatic right-sided intraorbital meningioma in a 4-year-old boy with proptosis and visual loss as presenting symptom. **b** Meningioma of the craniocervical transition in a 10-year-old NF2 patient. **c**, **d** Jugular tubercle meningioma with craniocervical extension along the right vertebral artery which was symptomatic by dysphagia due to paresis of the hypoglossal and glossopharyngeal nerve with atrophy of the right tongue in a 8-year-old girl

NF2 suspicion should always be raised in children with non-VS-related neurological symptoms attributed to other NF2-associated tumors including intracranial or spinal meningiomas (examples are illustrated in Fig. 3), non-VS schwannomas, or peripheral schwannomas. Especially since sporadic meningiomas in children are extremely rare [18] while frequent in NF2 [17, 19], they can be regarded as clear indiction for a suspicion of NF2 and should be prompted by further investigations like high-resolution MRI of the posterior fossa to detect very small intracanalicular VS, spinal MRI to detect schwannomas or ependymomas, detailed skin examination, and ophthalmological consultation.

Other features such as “isolated occurrence” of spinal intradural, paraspinal or peripheral nerve sheath tumors (schwannomas, not neurofibromas), or intramedullary ependymomas [6, 19] may also cause symptoms earlier than VS. Therefore, such occurrence should as well be considered as indication for a NF2 surveillance.

In general, children with a positive family history are diagnosed earlier [17]. To our surprise, a positive family history in our cohort was associated with a delayed diagnosis of about 1 year to children without a family history, though the difference was not statistically significant. Seven out of the 13 patients with a family history were registered in NF2 referral centers but did not follow the recommended monitorings nor the recommendation to screen the asymptomatic children.

This highlights the fact that despite or perhaps because of the experience of one parent with the problems, disadvantages, and negative psychological effects of coping with a life-changing burden of a non-curable genetic disease, children are not monitored early in life. Since NF2 patients are intellectually fully functional and not different from the general population, we suspect that rather the second mechanism of wanting to protect the children as along as possible from the shadow NF2 will casts on their life, makes parents to ignore the recommendations of early MRI and hearing monitoring at an age of perhaps 6–8 years, when children can cope with a MRI without sedation and would be compliant enough for hearing tests. Although this is understandable from the protective motivation all parents carry regarding their children, it seems necessary that NF centers in counseling their patients should spend more time on counseling regarding the children of their patients.

The positive mutation rate of over 90% in our pediatric cohort is very high, compatible to that known in familial cases [20]. For comparison, the detection rate was 51% in unselected NF2 patients without family history in the same previous study [20]. This high rate in our cohort may reflect the low frequency of mosaicism in patients with early onset of the disease. This is because mosaicism shifts the clinical spectrum of NF2 toward the mild end and consequently late onset of symptoms is a key parameter for mild phenotypes. The high rate of identifying a mutation provides a strategy for cases presenting non-specific symptoms of NF2 but not yet fulfilling the classical diagnostic criteria. Therefore, parents can be counseled or send to genetic counseling in case of tumor manifestations typical for NF2 or the indetification of a typical skin schwannoma.

A truncating *NF2* mutation is the ultimate evidence for the diagnosis of NF2 [21]. However, it should be emphasized that the non-detection of a mutation is not an evidence against the diagnosis. For such cases, extensive clinic and radiological examinations are essential.

Due to the rare occurrence of NF2 in the populations and the frequent unspecific early symptoms such as ophthalmological and cutaneous abnormalities, the diagnosis of NF2 in children remains challenging. Consequently, extensive clinical experience and expertise in the field of NF2 play an important role in this process. It is therefore advisable to refer suspicious cases to specialized centers or specialists where the patients receive comprehensive examination and interdisciplinary consideration covering multiple aspects including genetics, oncology, radiology, ophthalmology, dermatology, neurology, and off course hearing. Overlooking and underestimating non-specific symptoms in children may lead to relevant delays in diagnosing NF2 with the consequent of wasting valuable time for early medical and surgical treatment to protect the patients of the NF2 effects as long as possible. This particularly important for the management of bilateral vestibular schwannomas since early and close monitoring of the tumor growth and hearing as well as an intervention at an “ideal time” is the basis for better chances of hearing preservation.

## Data Availability

All data is included in this work.
